# Unpacking the cost implications of diagnosis-related groups reform for lumbar disc herniation patients in Chinese medicine: a closer look at evidence from China

**DOI:** 10.3389/fpubh.2025.1631872

**Published:** 2025-09-19

**Authors:** Meng-en Chen, Yu-han Wang, Jing Yu, Shi-ji Xia, Xiao-xi Zhang, Yan Wang, You-shu Yuan, Xuan Jia, He-nong Sun, Guo-ping Wu, Jia-yi Wang, Tian-zhen Cong, Fan-xin Kong, Hao-jia Hou, Jing-yu Yang, Zhi-wei Wang

**Affiliations:** ^1^School of Traditional Chinese Medicine, Beijing University of Chinese Medicine, Beijing, China; ^2^School of Management, Beijing University of Chinese Medicine, Beijing, China; ^3^Guang’anmen Hospital, China Academy of Chinese Medicine Sciences, Beijing, China; ^4^School of Health Management, Gansu University of Chinese Medicine, Lanzhou, China; ^5^Gansu Health and Population Development Research Center, Gansu Provincial Health and Wellness Commission, Lanzhou, China; ^6^School of Public Health, Lanzhou University, Lanzhou, China; ^7^National Institute of Chinese Medicine Development and Strategy, Beijing University of Chinese Medicine, Beijing, China

**Keywords:** LDH, DRG, TCM, hospitalization cost, Chinese medicine hospitals

## Abstract

**Objectives:**

Lumbar disc herniation (LDH) presents a significant economic burden globally, worsening in China due to an aging population. Traditional Chinese Medicine (TCM) offers effective treatment options for LDH, making its integration with Diagnosis-Related Groups (DRG) payment reform crucial for reducing medical costs and enhancing healthcare quality.

**Methods:**

We analyzed data from hospitalized patients at Qingyang City Hospital of Chinese Medicine, Gansu Province, from 2017 to 2022. Univariate analysis was conducted to examine changes in patient demographics before and after the DRG reform. A single group interrupted-time series analysis (ITSA) model was used to compare key indicators of medical costs and length of stay pre- and post-reform.

**Results:**

A total of 2,857 LDH patients were included in the study. Pre-DRG reform, 1,294 patients were recorded, with males comprising 46.06% and a mean age (SD) of 58.29 (14.22) years. Post-DRG reform, 1,563 patients were observed, with males accounting for 40.88% and a mean age (SD) of 60.64 (14.25) years. No significant differences were found in nationality, marital status, use of Chinese medicine diagnostic and therapeutic equipment, use of Chinese medicine diagnostic and treatment techniques or diagnosis and treatment based on Chinese medicine evidence (*p* > 0.05). However, significant differences were noted in sex, age, visit times, admission pathways, admission disease status, complications and comorbidities, and surgeries and operations (*p* < 0.05). Healthcare-related costs and length of stay are associated with sex, age, visit times, and other factors, showing a positive correlation among different costs and length of stay (*p* < 0.05). Post-reform, average monthly hospitalization cost decreased by CNY 36.78 ($\beta_{1}+\beta_{3}=-36.78,$*p* < 0.05), Chinese medicine cost fell by CNY 8.87 ($\beta_{1}+\beta_{3}=-8.87,$*p* < 0.05), and Western medicine cost dropped by CNY 31.68 ($\beta_{1}+\beta_{3}=-31.68,$*p* < 0.05). While the rising trend in diagnosis cost was curtailed, both medical services cost and TCM treatment cost increased, with the length of stay remaining stable.

**Conclusion:**

The DRG reform is associated with lower hospitalization cost and reduced costs for both Chinese medicine and Western medicine. However, its impact on overall medical services cost, diagnosis cost, TCM treatment cost, and length of stay is limited. Future DRG reform should leverage the distinctive advantages of TCM, enhance the payment system, improve treatment outcomes, and further reduce healthcare costs while shortening hospitalization times.

## 1 Introduction

Low back pain is one of modern society’s most common orthopedic conditions (1). Currently, over 600 million people suffer from low back pain worldwide, and this number is projected to exceed 800 million by 2050 (2–4). Low back pain attributable solely to occupational ergonomic factors contributed to global economic losses of US$216.1 billion. Recent Chinese healthcare statistics revealed an average hospitalization cost of ¥11,085.9 (US$1,604) per low back pain episode. Both China and the global community face substantial economic burdens from low back pain, with China experiencing particularly accelerated growth in this burden due to its rapidly aging population (5, 6). Low back pain ranks among the top causes of disability globally, with long recovery times that impose a significant economic burden on patients and their families. LDH has emerged as a primary contributor to low back pain, leg pain, and mobility impairments (7, 8), with its incidence climbing each year due to an aging population, evolving work patterns, lifestyle shifts, and increasingly sedentary behavior (9). Research from countries such as the United States (10, 11), the United Kingdom (12), Brazil (13), New Zealand (14), and Spain underscores the substantial healthcare burden posed by LDH (15, 16). Given China’s large population and accelerating aging process, healthcare costs associated with LDH are expected to continue rising soon.

TCM demonstrates unique advantages in treating LDH. It utilizes various modalities, including acupuncture, tuina (Chinese massage), herbal medicine, and moxibustion. By adopting a holistic approach and differential diagnosis, TCM effectively alleviates pain and enhances the body’s self-repair capabilities (17, 18). Furthermore, exercises such as Tai Chi and Wuqinxi have demonstrated significant effectiveness in preventing low back pain, as highlighted in several studies (19, 20). In contrast, conventional treatments like medication and surgery often carry numerous complications and side effects (21–23). TCM’s conservative, non-surgical methods typically result in fewer complications and better recovery outcomes (24–27). Reforming the DRG payment system in Chinese medicine hospitals to harness TCM strengths can significantly reduce hospitalization cost and length of stay for LDH patients, making this an important practical consideration.

DRG introduced by Fetter et al. in the 1980s, classify cases based on similarities in clinical processes and resource consumption (28). DRG has been adopted by various countries (29–33) for its benefits in hospital management and healthcare costs, achieving notable results (30, 34–36). Through decades of practice-oriented research and development, China has established several regional pilot DRG systems, including BJ-DRG, CN-DRG, CR-DRG, and C-DRG. These initiatives aim to address China’s rapidly growing healthcare expenditures while positively impacting the quality of medical services. On June 18, 2020, the China National Healthcare Security Administration officially promulgated the “China Healthcare Security Diagnosis Related Groups (CHS-DRG) Fine Classification Scheme (Version 1.0),” initiating nationwide pilot implementation and subsequent progressive rollout. The CHS-DRG framework adopts China’s national health insurance versions of ICD-10 (comprising 2,048 diagnostic categories, 10,172 subcategories, and 33,324 specific entries) and ICD-9-CM-3 (including 890 surgical procedure subcategories, 3,666 detailed items, and 12,995 entries). Following the grouping principles of “clinical process similarity” and “resource consumption similarity,” the system established 376 Adjacent Diagnosis-Related Groups (ADRGs), consisting of 167 surgical procedure groups, 22 non-operating room procedure groups, and 187 medical diagnosis groups, comprehensively covering all acute and severe short-term hospitalization cases (37, 38). While traditional Chinese medicine hospitals are required to implement the unified CHS-DRG standards alongside other medical institutions, a gradual transition period is permitted. For certain TCM-dominant disease categories with demonstrated treatment efficacy and stable cost structures, specially designated reimbursement standards may be alternatively applied. As a vital part of healthcare in China, the reform of DRG payment systems in Chinese medicine hospitals is important, particularly for LDH, a condition where TCM shows distinct advantages.

This study focuses on LDH patients at Qingyang City Hospital of Chinese Medicine, Gansu Province, examining the impact of DRG payment reform on healthcare-related costs and length of stay. The findings aim to provide insights for Chinese health policymakers to advance DRG reforms, leveraging TCM’s strengths to alleviate the economic burden of diseases like LDH in China.

## 2 Materials and methods

### 2.1 Data sources

Data were sourced from the Gansu Provincial Health and Health Commission, covering case records from January 2017 to June 2022 for Qingyang City Hospital of Chinese Medicine. Inclusion criteria included TCM diagnosis code International Classification of Disease (ICD)-10 M51.202 and TCM coding BNS150 (1995 version) or A03.06.04.06.01 (2021 version). Exclusion criteria involved length of stay less than 1 day or greater than 90 days, zero hospitalization cost, and logically inconsistent visit information, resulting in a final dataset of 2,857 valid cases.

The data were regularly validated and cleaned by qualified personnel, ensuring reliable quality for analysis. The dataset encompasses patient demographics, including sex, age, and marital status, alongside medical information including visit times, surgeries and operations, complications and comorbidities, medical costs, and length of stay.

To evaluate the impact of DRG reform on medical costs and length of stay for LDH patients in Chinese medicine hospitals, our study employed a quasi-experimental model for data analysis. As one of the pioneering tertiary Chinese medicine hospitals in Northwest China to implement DRG payment reform, this research is instrumental in advancing DRG reimbursement systems in the TCM sector.

### 2.2 Statistical analysis

To address healthcare economic costs, we used 2016 as the base year and adjusted relevant costs based on the Consumer Price Index (CPI) for healthcare in Gansu Province from 2017 to 2022, minimizing potential biases in the study. It is worth highlighting that Qingyang City in Gansu Province, began trial implementation of the DRG reimbursement system in October 1, 2019. Accordingly, the period from January 1, 2017 to September 30, 2019 is designated as the pre-reform phase, during which the medical insurance payment was implemented under the fee-for-service (FFS) model. In contrast, the period from October 1, 2019 to June 30, 2022 is defined as the post-reform phase, with the implementation of the DRG-based medical insurance payment method.

Next, we compared the basic characteristics of patient visits before and after the DRG reform. For normally distributed numerical variables, paired sample *t*-tests were used, reporting data as means and standard deviations. For non-normally distributed numerical variables, we applied the Wilcoxon rank-sum test, expressing results in median and quartiles. Categorical variables were analyzed using chi-square tests, with frequencies and percentages reported. Moreover, multiple linear regression and Spearman correlation analyses were performed to investigate determinants of hospitalization expenditures and length of stay, as well as inter-variable relationships.

Finally, we employed an interrupted time-series analysis (ITSA) model - a quasi-experimental method specifically designed to assess causal effects of interventions (e.g., policy changes, clinical practice implementations) on longitudinal data. This approach compares pre- versus post-intervention trends while controlling for underlying temporal patterns, thereby providing robust effect estimation (39–41). We applied ITSA to evaluate key healthcare cost indicators for LDH patients in Chinese medicine hospitals. The analysis included hospitalization cost, medical service cost, diagnostic cost, TCM treatment cost, Chinese medicine cost, Western medicine cost, and length of stay. This methodology facilitated a comprehensive investigation of the changes in these indicators before and after the DRG reform, with the model equation expressed as follows:


$$\;Y_{t}=\beta_{0}+\beta_{1}T_{t}+\beta_{2}X_{t}+\beta_{3}X_{t}T_{t}+\varepsilon_{t}$$

**Figure 1:**
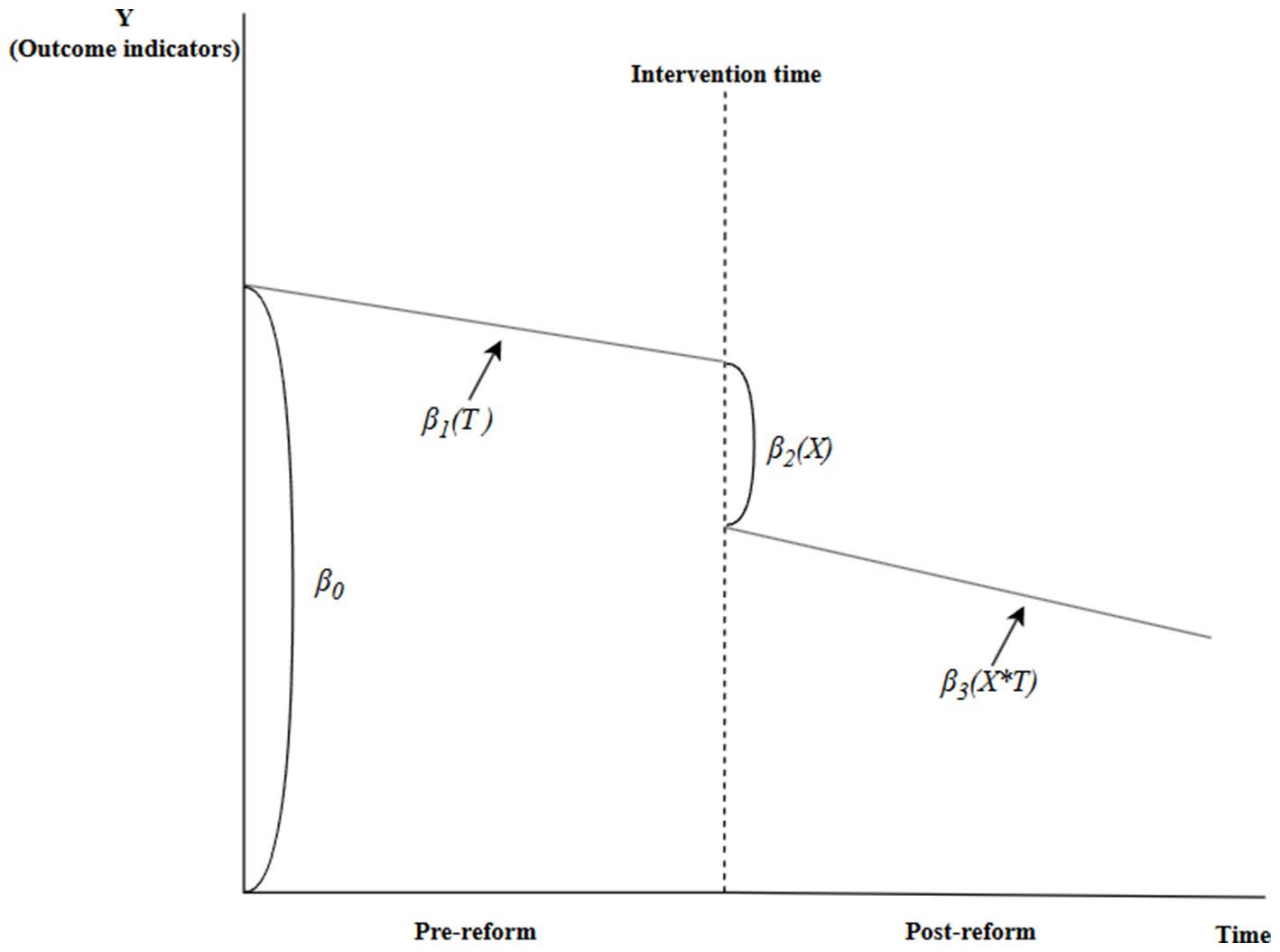
Schematic diagram of interrupted time-series model.

In this equation, $Y_{t}$ is the dependent variable (the primary outcome), β_0_ is the intercept, β_1_ reflects the pre-reform trend, β_2_ indicates the level change at the reform, and β_3_ represents the difference in slopes between the post-reform and pre-reform periods, with the post-reform slope being the sum of β_1_ and β_3_. $T_{t}$ denotes the time series spanning January 2017 to June 2022, with T ranging from 1 to 66. Interaction terms include $X_{t}T_{t}$,$\;{\mathrm{ZT}}_{t}$, ${\mathrm{ZX}}_{t}$, and ${\mathrm{ZX}}_{t}T_{t}$, while $\varepsilon_{t}$ denotes random error. To address the skewed distributions of healthcare costs and length of stay, we selected the median values for each month as the data for the interrupted time series analysis, ensuring the rigor of the analysis. A schematic of the model is shown in Figure 1.

We used the Cumby-Huizinga tests to assess the autocorrelation of the dependent variable and adjusted calculations using the ‘lag (#)’ command along with the Newey-West method (39, 40, 42). All statistical analyses were conducted using Excel 2019, SPSS 26.0, and Stata 15.0, with a significance level set at *α* = 0.05.

## 3 Results

### 3.1 General information of LDH hospitalized patients in Qingyang City Hospital of Chinese medicine in pre- and post-DRG reform

We included a total of 2,857 LDH patients in our analysis. Before the implementation of the DRG reform, there were 1,294 cases, with 46.06% of the patients being male and an average age (SD) of 58.29 (14.22) years. Following the DRG reform, the sample comprised 1,563 cases, with a male proportion of 40.88% and an average age (SD) of 60.64 (14.25) years. Further demographic details are provided in Table 1.

**Table 1:** General information on LDH patients pre- and post-DRG reform.

Items	DRG^1^ reform		*χ*^2^*/t*/*Z*-value	*p*-value
	Pre-reform (*n* = 1,294)	Post-reform (*n* = 1,563)		
Sex (Male/n, %)^a^^,^^j^	596(46.06)	639(40.88)	7.728	0.005
Age(years)^b^	58.29 ± 14.22	60.64 ± 14.25	−4.257	<0.001
Nationality (Han/n, %)^c^^,^^j^	1,292(99.85)	1,559(99.74)	0.032	0.858
Visit times (One time /n, %)^d^^,^^j^	1,277(98.69)	1,400(89.57)	99.629	<0.001
Marital status (Married/n, %)^e^^,^^j^	1,147(88.64)	1,401(89.64)	0.727	0.394
Admission pathways (Outpatient or emergency/n, %)^f^^,^^j^	255(19.71)	35(2.24)	236.820	<0.001
Admission disease status (Determination/n, %)^g^^,^^j^	1,252(96.75)	1,539(98.46)	9.175	0.002
Complications and comorbidities (Yes/n, %)^h^^,^^j^	50(3.86)	684(43.76)	590.282	<0.001
Use of Chinese medicine diagnostic and therapeutic equipment (Yes/n, %)^h^^,^^j^	1,047(80.91)	1,246(79.72)	0.636	0.425
Use of Chinese medicine diagnostic and treatment techniques (Yes/n, %)^h^^,^^j^	1,045(80.76)	1,239(79.27)	0.976	0.323
Diagnosis and treatment based on Chinese medicine evidence (Yes/n, %)^h^^,^^j^	1,101(85.09)	1,320(84.45)	0.219	0.640
Surgeries and operations (Yes/n, %)^h^^,^^j^	98(7.57)	776(49.65)	590.216	<0.001
Hospitalization cost (CNY)^2^^,^^i^	5712.74(4273.84,7428.76)	5361.70(4051.83,7425.91)	−0.957	0.339
Medical services cost (CNY)^2^^,^^i^	691.36(429.18,1085.52)	1023.30(720.79,1409.90)	−13.195	<0.001
Diagnosis cost (CNY)^2^^,^^i^	1375.20(955.61,1925.81)	1454.59(1151.55,1961.62)	−2.522	0.012
TCM^3^ treatment cost (CNY)^2^^,^^i^	1222.40(698.67,2149.66)	2089.49(1106.24,4027.23)	−15.377	<0.001
Chinese medicine cost (CNY)^2^^,^^i^	494.80(233.01,869.84)	436.22(239.01,738.85)	−2.794	0.005
Western medicine cost (CNY)^2^^,^^i^	1355.00(873.14,2004.53)	602.36(278.47,1210.06)	−16.392	<0.001
Length of stay (days)^2^^,^^i^	11.00(8.00,15.00)	10.00(8.00,14.00)	−3.550	<0.001

1 DRG, diagnosis-related group.

2 CNY, Chinese Yuan.

3 TCM, traditional Chinese medicine.

a Sex: male vs. female.

b The normal distribution continuous data were presented as “mean ± standard deviation,” the data were compared using paired-samples t-test (t).

c Nationality: Han vs. other nationalities.

d Visit times: one time vs. two or more times.

e Marital status: married vs. unmarried and others.

f Admission pathways: outpatient or emergency vs. other pathways.

g Admission disease status: determination vs. indetermination or none.

h Categorization outcome includes yes and no.

i The non-normal distribution continuous data were presented as “median (the first quartile, the third quartile),” the data were compared using the Wilcoxon rank sum test (Z).

j The categorical data were presented as numbers (frequencies, %), and the chi-square test was used for categorical data (χ^2^).

**Figure 2:**
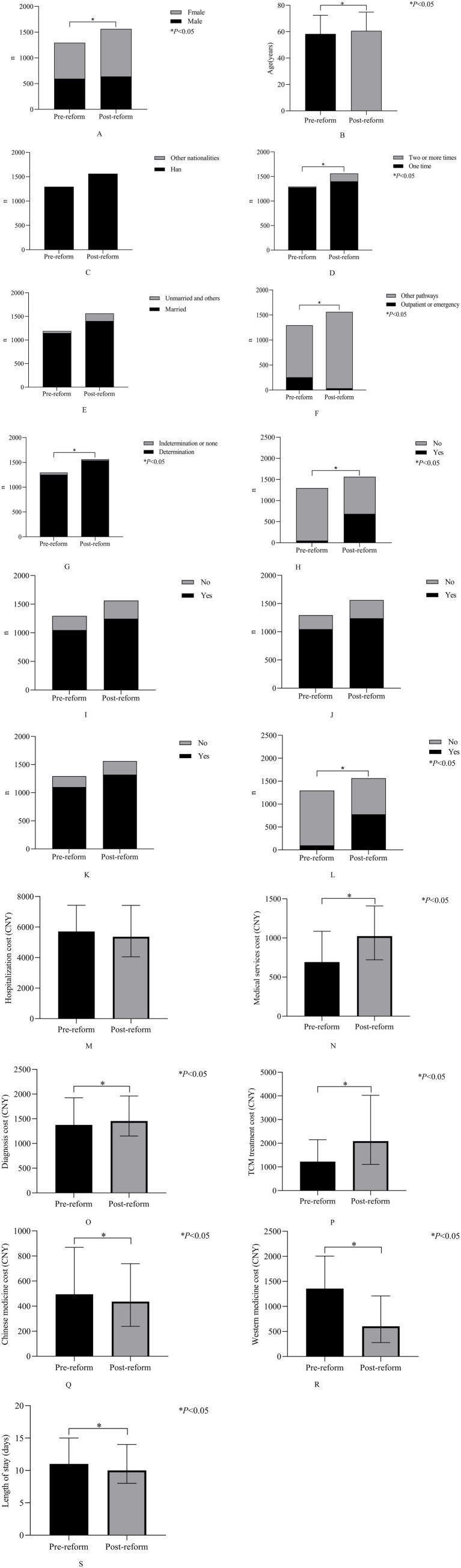
Comparative analysis of LDH patients characteristics pre- and post-DRG reform.

As shown in Table 1 and Figure 2, there were no statistical differences in nationality, marital status, use of Chinese medicine diagnostic and treatment techniques, use of Chinese medicine diagnostic and treatment techniques, diagnosis and treatment based on Chinese medicine evidence, and hospitalization cost among LDH patients pre- and post-DRG reforms (*p* > 0.05), but there were significant differences in sex, age, visit times, admission pathways, admission disease status, complications and comorbidities, surgeries and operations, medical services cost, diagnosis cost, TCM treatment cost, Chinese medicine cost, Western medicine cost and length of stay among LDH patients pre- and post-DRG reforms (*p* < 0.05).

### 3.2 Results of factors affecting LDH patients’ healthcare-related costs and length of stay in Qingyang City Hospital of Chinese medicine

To further investigate the factors influencing healthcare-related costs and length of stay in LDH patients, we constructed multiple linear regression models using hospitalization cost, medical service cost, diagnostic cost, TCM treatment cost, Chinese medicine cost, and Western medicine cost, and length of stay as dependent variables, with sex, age, nationality, and other general information as independent variables. Stepwise regression was employed for the analysis.

The regression results (Table 2) revealed that: hospitalization cost was significantly lower in patients using Chinese medicine diagnostic and therapeutic equipment (*p* < 0.05); medical service cost increased with complications, two or more times hospitalizations, use of Chinese medicine diagnostic and treatment technique, and post-DRG reform (*p* < 0.05); diagnostic cost decreased for patients with complications, younger patients, outpatient or emergency admissions, use of Chinese medicine diagnostic and therapeutic equipment, and no diagnosis and treatment based on Chinese medicine evidence (*p* < 0.05); TCM treatment cost was higher for complicated cases, females, two or more times hospitalizations, other pathways, determination disease status, use of Chinese medicine diagnostic and treatment techniques, and pre-DRG reform (*p* < 0.05); Chinese medicine cost decreased for uncomplicated cases, males, younger patients, no use of Chinese medicine diagnostic and treatment techniques, and post-DRG reform (*p* < 0.05); Western medicine cost increased for uncomplicated cases, older adults patients, one time hospitalizations, outpatient or emergency admissions, indetermination or none disease status, no use of Chinese medicine diagnostic and therapeutic equipment, and pre-DRG reform (*p* < 0.05); and length of stay was shorter for uncomplicated cases, older adults patients, one time hospitalizations, use of Chinese medicine diagnostic and therapeutic equipment, no use of Chinese medicine diagnostic and treatment techniques, and post-DRG reform (*p* < 0.05).

**Table 2:** Results of multiple linear regression analysis for healthcare-related costs and length of stay in LDH patients.

Variables	Hospitalization cost	Medical services cost	Diagnosis cost	TCM treatment cost	Chinese medicine cost	Western medicine cost	Length of stay
Constant	5001.91 (9.52)^*^	866.43 (11.42)^*^	422.92 (3.65)^*^	238.83 (1.10)	380.84 (6.98)^*^	1361.79 (5.26)^*^	12.65 (14.70)^*^
Complications and comorbidities (ref = No)		181.18 (4.918)^*^	−87.30 (−2.46)^*^	788.68 (14.66)^*^	65.99 (2.77)^*^	−159.83 (−2.52)^*^	2.04 (5.99)^*^
Sex(ref = Male)				92.07 (2.22)^*^	37.83 (2.04)^*^		
Age			3.31 (3.10)^*^		4.88 (7.53)^*^	4.53 (2.63)^*^	−0.02 (−2.29)^*^
Visit times (ref = One time)		131.37 (2.21)^*^		537.66 (6.21)^*^		−215.83 (−2.11)^*^	2.19 (3.98)^*^
Admission pathways (ref = Outpatient or emergency)			363.56 (7.07)^*^	659.69 (9.17)^*^		−323.04 (−3.81)^*^	
Admission disease status (ref = Determination)				−279.92 (−2.00)^*^		407.79 (2.47)^*^	
Use of Chinese medicine diagnostic and therapeutic equipment (ref = Yes)	2281.12 (5.48)^*^		431.27 (6.70)^*^			414.30 (6.50)^*^	2.34 (2.36)^*^
Use of Chinese medicine diagnostic and treatment techniques (ref = Yes)		−113.43 (−3.18)^*^		−403.90 (−7.52)^*^	−82.39 (−3.55)^*^		−3.15 (−3.19)^*^
Diagnosis and treatment based on Chinese medicine evidence (ref = Yes)			−179.56 (−2.53)^*^				
DRG reform (ref = Pre-reform)		206.52 (6.42)^*^		−398.25 (−8.25)^*^	−107.65 (−5.18)^*^	−663.61 (−11.65)^*^	−1.86 (−6.26)^*^

*t-*values are shown in parentheses. ^*^p < 0.05.

To further elucidate the correlations between hospitalization-related costs and length of stay, we conducted Spearman correlation analyses. The results demonstrated statistically significant positive correlations (*p* < 0.05) among all measured variables, including hospitalization cost, medical service cost, TCM treatment cost, Chinese medicine cost, Western medicine cost, and length of stay, indicating consistent directional trends across these parameters. Complete results are presented in Table 3.

**Table 3:** Results of correlation analysis for healthcare-related costs and length of stay in LDH patients.

Variables	Hospitalization cost	Medical services cost	Diagnosis cost	TCM treatment cost	Chinese medicine cost	Western medicine cost	Length of stay
Hospitalization cost	1						
Medical services cost	0.687^*^	1					
Diagnosis cost	0.387^*^	0.075^*^	1				
TCM treatment cost	0.591^*^	0.363^*^	−0.034	1			
Chinese medicine cost	0.514^*^	0.464^*^	−0.058^*^	0.350^*^	1		
Western medicine cost	0.627^*^	0.274^*^	0.267^*^	0.107^*^	0.235^*^	1	
Length of stay	0.821^*^	0.724^*^	0.156^*^	0.583^*^	0.483^*^	0.495^*^	1

^*^p < 0.05.

### 3.3 Results of ITSA of DRG reform on LDH patients’ healthcare-related costs in Qingyang City Hospital of Chinese medicine

We performed Cumby-Huizinga autocorrelation tests on hospitalization cost, medical service cost, diagnostic cost, TCM treatment cost, Chinese medicine cost, and Western medicine cost. The results indicated that hospitalization cost, TCM treatment cost, and Chinese medicine cost exhibited no autocorrelation. However, diagnostic cost and Western medicine cost may demonstrate first-order autocorrelation, while medical service cost could indicate second-order autocorrelation, as summarized in Table 4. To ensure the robustness of our interrupted time-series analysis, we employed the ‘lag (1)’or ‘lag (2)’ command to adjust for autocorrelation effects in the medical costs analysis.

**Table 4:** Autocorrelation test results of healthcare-related costs for LDH patients.

Items	*H*_0_: q = 0 (serially uncorrelated)				*H*_0_: q = lag-1				Items	*H*_0_: q = 0 (serially uncorrelated)				*H*_0_: q = lag-1			
	*H*_1_: s.c. present at range specified				*H*_1_: s.c. present at lag specified					*H*_1_: s.c. present at range specified				*H*_1_: s.c. present at lag specified			
	lags	chi2	df	*p*-value	lags	chi2	df	*p*-value		lags	chi2	df	*p*-value	lags	chi2	df	*p*-value
Hospitalization cost	1–1	0.521	1	0.471	1	0.521	1	0.471	Medical services cost	1–1	9.548	1	0.002	1	9.548	1	0.002
	1–2	1.212	2	0.545	2	0.548	1	0.459		1–2	11.001	2	0.004	2	4.081	1	0.043
	1–3	2.381	3	0.497	3	0.762	1	0.383		1–3	11.044	3	0.012	3	1.324	1	0.250
	1–4	3.927	4	0.416	4	0.741	1	0.390		1–4	11.183	4	0.025	4	0.163	1	0.686
	1–5	4.044	5	0.543	5	0.753	1	0.386		1–5	11.200	5	0.048	5	0.006	1	0.938
	1–6	5.550	6	0.475	6	0.809	1	0.369		1–6	12.200	6	0.058	6	0.900	1	0.343
Diagnosis cost	1–1	7.124	1	0.008	1	7.124	1	0.008	TCM^1^ treatment cost	1–1	0.006	1	0.939	1	0.006	1	0.939
	1–2	7.201	2	0.027	2	0.964	1	0.326		1–2	1.578	2	0.454	2	1.576	1	0.209
	1–3	8.017	3	0.046	3	1.153	1	0.283		1–3	1.585	3	0.663	3	0.021	1	0.886
	1–4	8.069	4	0.089	4	0.438	1	0.508		1–4	4.990	4	0.288	4	2.536	1	0.111
	1–5	8.089	5	0.151	5	0.087	1	0.768		1–5	5.025	5	0.413	5	0.021	1	0.884
	1–6	8.090	6	0.232	6	<0.001	1	0.993		1–6	5.044	6	0.538	6	0.149	1	0.699
Chinese medicine cost	1–1	1.161	1	0.281	1	1.161	1	0.281	Western medicine cost	1–1	8.915	1	0.003	1	8.915	1	0.003
	1–2	2.047	2	0.359	2	0.610	1	0.435		1–2	9.248	2	0.010	2	2.103	1	0.147
	1–3	2.220	3	0.528	3	0.026	1	0.873		1–3	11.531	3	0.009	3	0.294	1	0.588
	1–4	2.464	4	0.651	4	0.086	1	0.769		1–4	11.976	4	0.018	4	1.050	1	0.305
	1–5	3.616	5	0.606	5	0.697	1	0.404		1–5	11.980	5	0.035	5	0.859	1	0.354
	1–6	4.259	6	0.642	6	0.119	1	0.730		1–6	20.100	6	0.003	6	7.127	1	0.008

1 TCM, traditional Chinese medicine.

**Figure 3:**
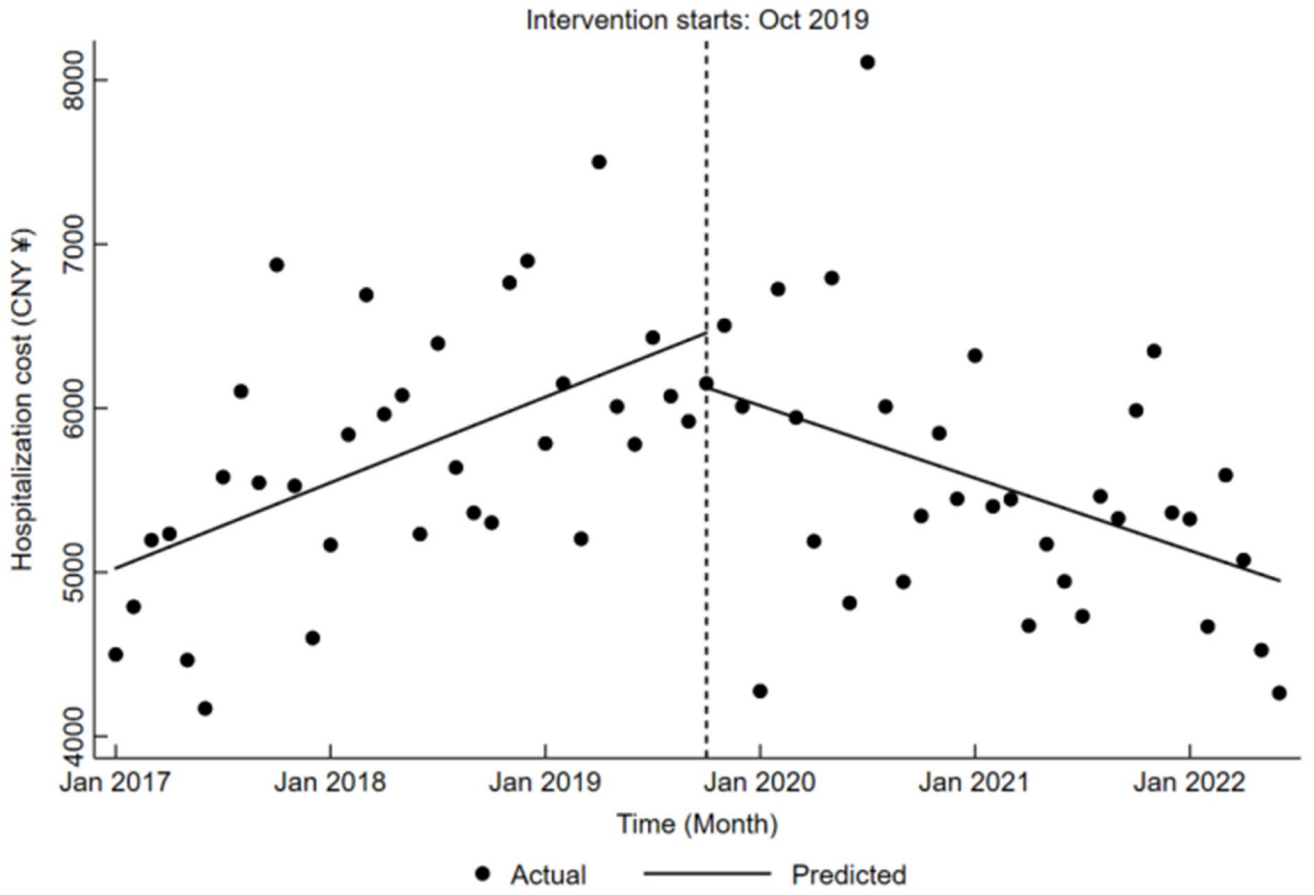
Trends in hospitalization cost for LDH patients pre- and post-DRG reform.

Before the DRG reform at Qingyang City Hospital of Chinese Medicine, hospitalization cost exhibited a significant upward trajectory, with an average monthly increase of CNY 43.49 (*β*₁ = 43.49, *p* < 0.05). Conversely, during the reform, fluctuations in hospitalization cost were not statistically significant (*β*₂ = −333.52, *p* > 0.05). Following the reform, however, cost demonstrated a pronounced downward trend, decreasing by an average of CNY 36.78 per month (*β*₁ + *β*₃ = −36.78, *p* < 0.05). Comprehensive results are presented in Table 5, and the corresponding trend changes are illustrated in Figure 3.

**Table 5:** Results of ITS analysis on healthcare-related costs for LDH patients.

Items	Value	Std. Err.	*t*-value	*p*-value	95% Conf. interval
Hospitalization cost (CNY^2^)					
Baseline level, *β*_0_	5024.90	211.29	23.78	<0.001	4602.53 to 5447.27
Baseline trend, *β*_1_	43.49	10.97	3.97	<0.001	21.56 to 65.41
Level change, *β*_2_	−333.52	367.83	−0.91	0.368	−1068.81 to 401.77
Trend change, *β*_3_	−80.27	17.31	−4.64	<0.001	−114.87 to −45.67
Late trend, *β*_1_ + *β*_3_	−36.78	13.39	−2.75	0.008	−63.56 to −10.01
Medical services cost (CNY^2^)					
Baseline level, *β*_0_	833.54	127.17	6.55	<0.001	579.32 to 1087.76
Baseline trend, *β*_1_	−2.76	7.24	−0.38	0.704	−17.25 to 11.72
Level change, *β*_2_	361.98	160.06	2.26	0.027	42.02 to 681.95
Trend change, *β*_3_	−0.50	7.90	−0.06	0.950	−16.28 to 15.28
Late trend, *β*_1_ + *β*_3_	−3.26	2.83	−1.15	0.254	−8.93 to 2.40
Diagnosis cost (CNY^2^)					
Baseline level, *β*_0_	989.30	61.10	16.19	<0.001	867.15 to 1111.44
Baseline trend, *β*_1_	26.16	3.49	7.49	<0.001	19.18 to 33.13
Level change, *β*_2_	−307.11	87.34	−3.52	0.001	−481.70 to −132.52
Trend change, *β*_3_	−29.82	4.91	−6.07	<0.001	−39.64 to −19.99
Late trend, *β*_1_ + *β*_3_	−3.66	3.18	−1.15	0.254	−10.02 to 2.70
TCM^1^ treatment cost (CNY^2^)					
Baseline level, *β*_0_	709.14	93.72	7.57	<0.001	521.78 to 896.49
Baseline trend, *β*_1_	39.04	4.68	8.35	<0.001	29.70 to 48.39
Level change, *β*_2_	−466.54	196.34	−2.38	0.021	−859.01 to −74.07
Trend change, *β*_3_	11.03	11.89	0.93	0.357	−12.74 to 34.81
Late trend, *β*_1_ + *β*_3_	50.08	10.94	4.58	<0.001	28.21 to 71.94
Chinese medicine cost (CNY^2^)					
Baseline level, *β*_0_	587.73	59.24	9.92	<0.001	469.32 to 706.14
Baseline trend, *β*_1_	−3.96	2.59	−1.53	0.132	−9.15 to 1.22
Level change, *β*_2_	142.27	62.41	2.28	0.026	17.51 to 267.04
Trend change, *β*_3_	−4.91	3.36	−1.46	0.148	−11.63 to 1.80
Late trend, *β*_1_ + *β*_3_	−8.87	2.13	−4.16	<0.001	−13.14 to −4.61
Western medicine cost (CNY^2^)					
Baseline level, *β*_0_	1531.37	104.52	14.65	<0.001	1322.44 to 1740.29
Baseline trend, *β*_1_	−9.93	4.74	−2.10	0.040	−19.40 to −0.46
Level change, *β*_2_	−41.39	119.89	−0.35	0.731	−281.05 to 198.27
Trend change, *β*_3_	−21.75	6.32	−3.44	0.001	−34.38 to −9.12
Late trend, *β*_1_ + *β*_3_	−31.68	4.17	−7.59	<0.001	−40.02 to −23.34

1 TCM, traditional Chinese medicine.

2 CNY, Chinese Yuan.

**Figure 4:**
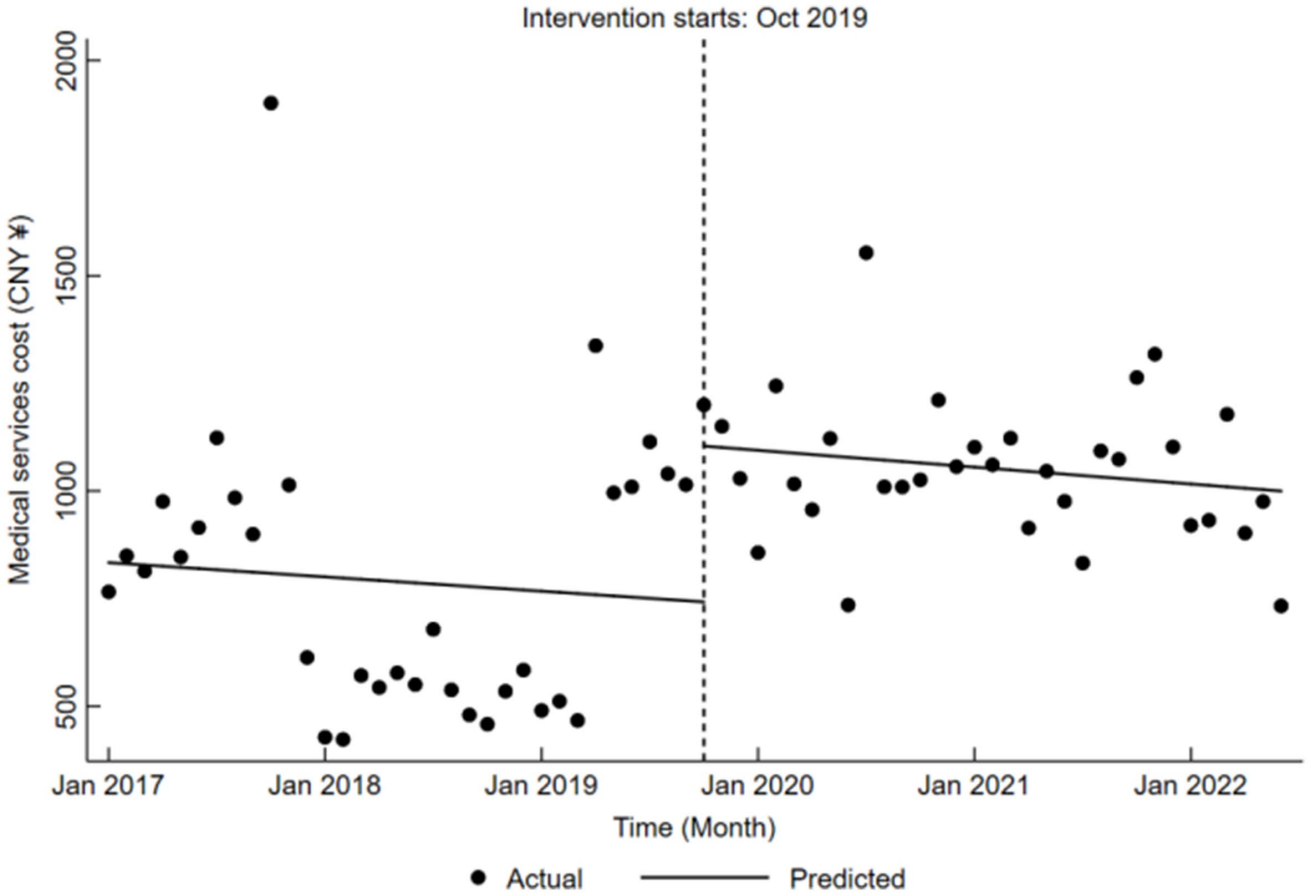
Trends in medical services cost for LDH patients pre- and post-DRG reform.

In terms of medical services cost, the pre-reform trend was not significant (*β*₁ = −2.76, *p* > 0.05). Nevertheless, during the reform, there was a substantial increase of CNY 361.98 (*β*₂ = 361.98, *p* < 0.05), while post-reform changes remained insignificant (*β*₁ + *β*₃ = −3.26, *p* > 0.05), as detailed in Table 5 and depicted in Figure 4.

**Figure 5:**
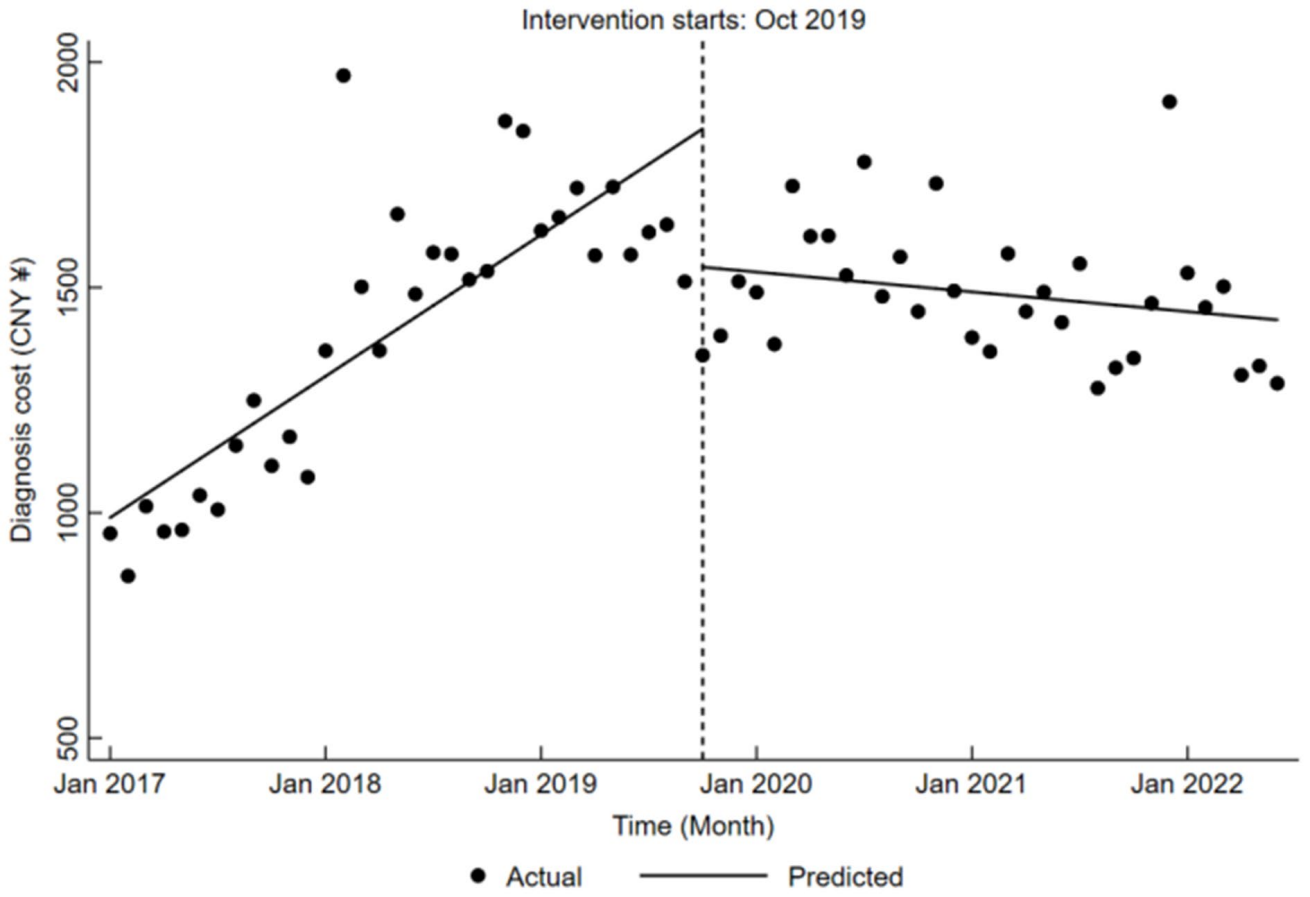
Trends in diagnosis cost for LDH patients pre- and post-DRG reform.

Regarding diagnosis cost, a clear upward trend was evident before the reform, with an average monthly increase of CNY 26.16 (*β*₁ = 26.16, *p* < 0.05). During the reform, diagnosis cost significantly decreased by CNY 307.11 (*β*₂ = −307.11, *p* < 0.05), yet the post-reform trend was again not significant (*β*₁ + *β*₃ = −3.66, *p* > 0.05), as outlined in Table 5 and shown in Figure 5.

**Figure 6:**
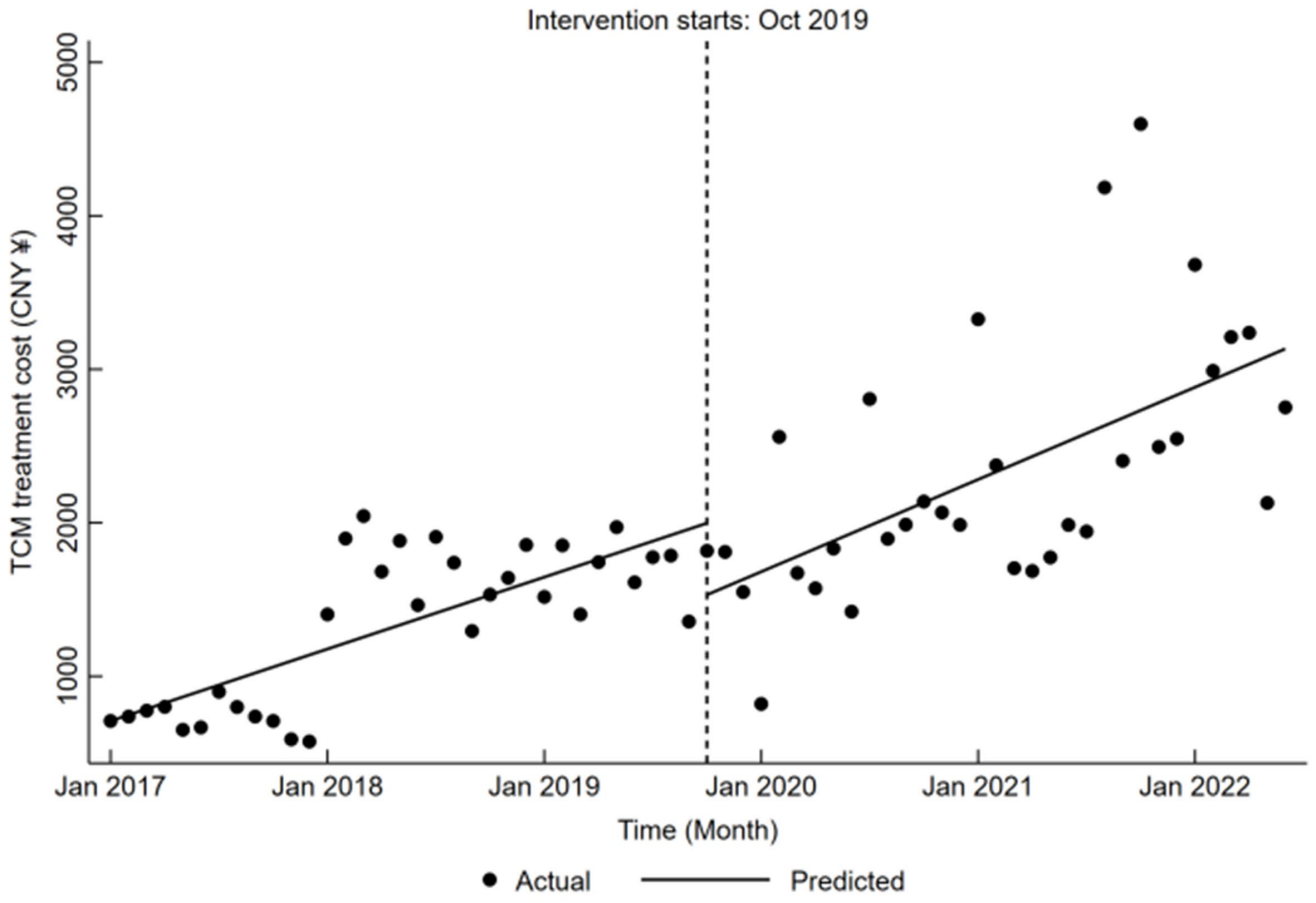
Trends in TCM treatment cost for LDH patients pre- and post-DRG reform.

Concerning TCM treatment cost, there was a notable increase before the reform, averaging CNY 39.04 per month (*β*₁ = 39.04, *p* < 0.05). Following a significant decrease of CNY 466.54 during the reform (*β*₂ = −466.54, *p* < 0.05), the cost rebounded, displaying a clear upward trend post-reform, with an average increase of CNY 50.08 (*β*₁ + *β*₃ = 50.08, *p* < 0.05), as detailed in Table 5 and represented in Figure 6.

**Figure 7:**
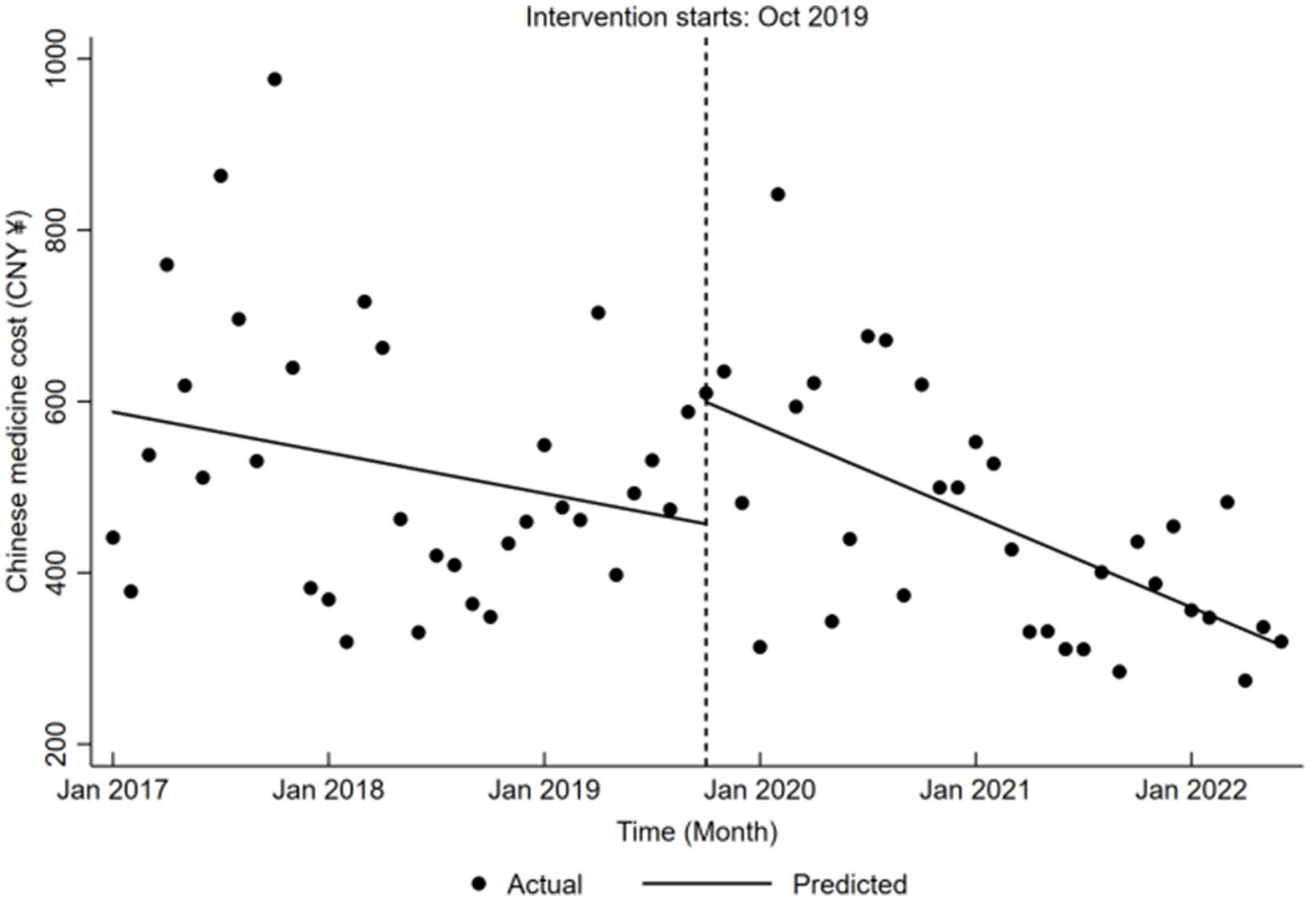
Trends in Chinese medicine cost for LDH patients pre- and post-DRG reform.

Chinese medicine cost exhibited no significant trend before the reform (*β*₁ = −3.96, *p* > 0.05). However, during the reform, the cost rose significantly by CNY 142.27 (*β*₂ = 142.27, *p* < 0.05), while post-reform expenses revealed a significant downward trend, decreasing by an average monthly of CNY 8.87 (*β*₁ + *β*₃ = −8.87, *p* < 0.05), as presented in Table 5 and illustrated in Figure 7.

**Figure 8:**
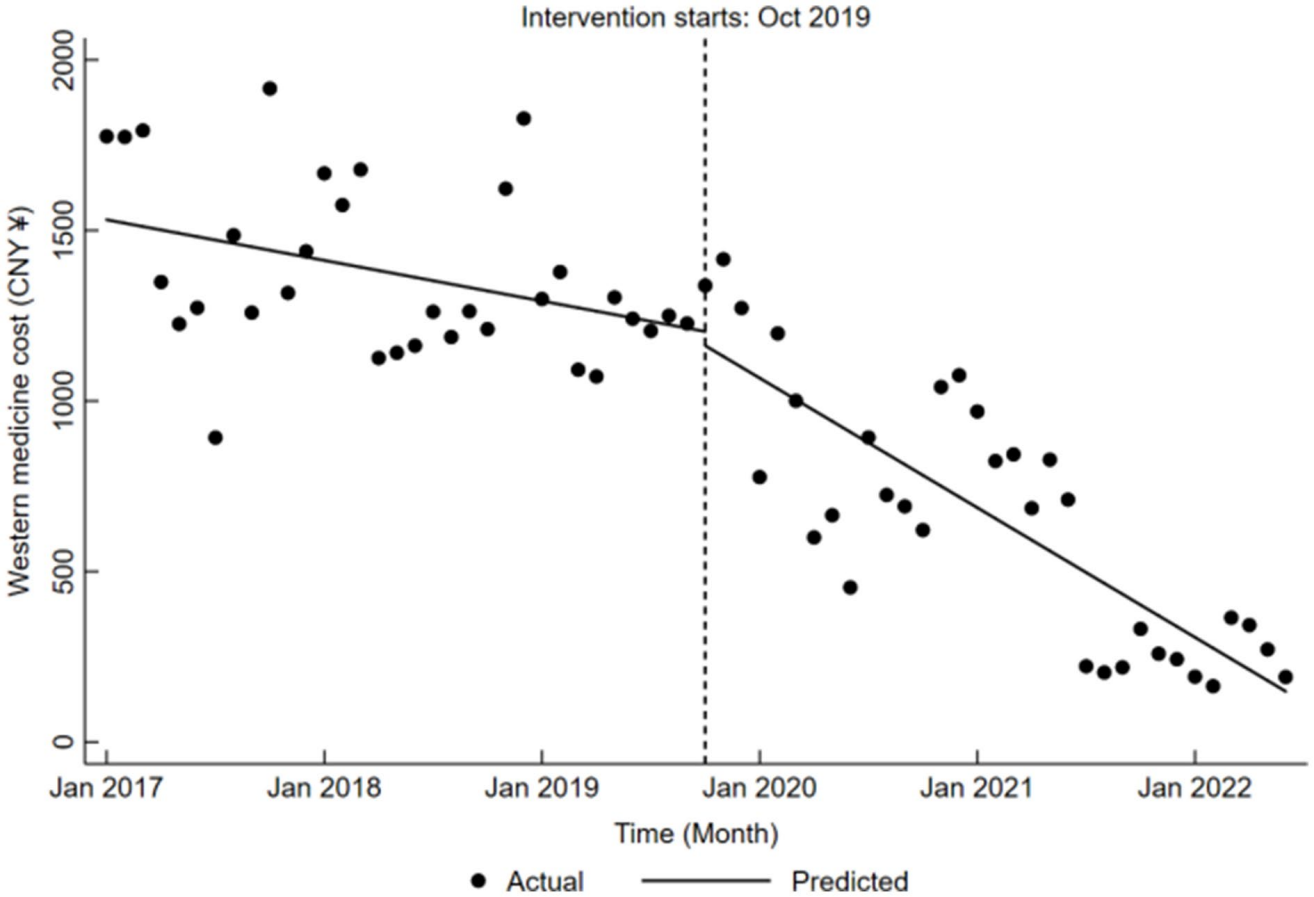
Trends in Western medicine cost for LDH patients pre- and post-DRG reform.

Finally, Western medicine cost showed a significant downward trend before the reform, decreasing by an average of CNY 9.93 (*β*₁ = −9.93, *p* < 0.05). Changes during the reform were not statistically significant (*β*₂ = −41.39, *p* > 0.05). However, the cost exhibited a marked downward trend, with an average monthly decrease of CNY 31.68 (*β*₁ + *β*₃ = −31.68, *p* < 0.05) post-reform, which was notably more pronounced than the pre-reform trend. Detailed results can be found in Table 5, with trend changes depicted in Figure 8.

### 3.4 Results of ITSA of DRG reform on length of stay in Qingyang City Hospital of Chinese medicine

We conducted a Cumby-Huizinga autocorrelation test on the length of stay, which revealed no evidence of autocorrelation, as presented in Table 6. This finding underscores the stability of the data regarding length of stay.

**Table 6:** Autocorrelation test results of length of stay for LDH patients.

*H*_0_: q = 0 (serially uncorrelated)				*H*_0_: q = lag-1			
*H*_1_: s.c. present at range specified				*H*_1_: s.c. present at lag specified			
lags	chi2	df	*p*-value	lags	chi2	df	*p*-value
1–1	0.159	1	0.690	1	0.159	1	0.690
1–2	0.250	2	0.882	2	0.072	1	0.789
1–3	0.649	3	0.885	3	0.338	1	0.561
1–4	0.986	4	0.912	4	0.245	1	0.621
1–5	0.988	5	0.964	5	0.025	1	0.874
1–6	8.317	6	0.216	6	6.733	1	0.010

Pre-DRG reform, the trend in length of stay was not statistically significant, with a coefficient of *β*₁is −0.03 (*p* > 0.05). This lack of significance indicates that there were no noteworthy changes in the average length of stay during this period.

**Figure 9:**
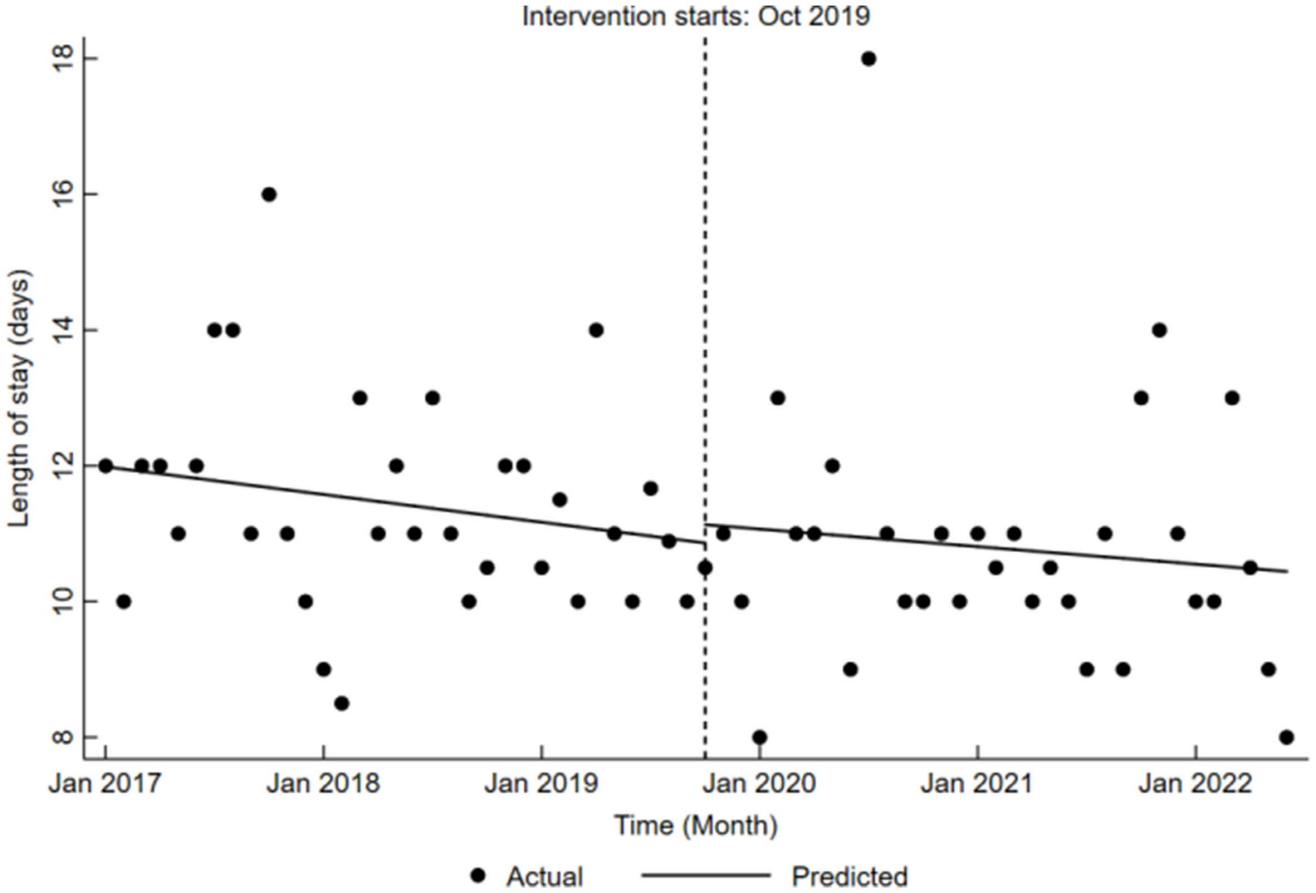
Trends in length of stay for LDH patients pre- and post-DRG reform.

Similarly, the analysis of length of stay during the reform period also yielded insignificant results (*β*₂ = 0.27, *p* > 0.05), and post-reform trends remained unremarkable, as reflected in the combined coefficient (*β*₁ + *β*₃ = −0.02, *p* > 0.05). Detailed results are provided in Table 7, and the corresponding trend changes are illustrated in Figure 9, further emphasizing the consistency of length of stay across the study periods.

**Table 7:** Results of ITS analysis on length of stay for LDH patients.

Item	Value	Std. Err.	*t*-value	*p*-value	95% Conf. interval
Baseline level, *β*_0_	11.99	0.54	22.21	<0.001	10.91 to 13.06
Baseline trend, *β*_1_	−0.03	0.02	−1.41	0.163	−0.08 to 0.01
Level change, *β*_2_	0.27	0.80	0.34	0.737	−1.33 to 1.87
Trend change, *β*_3_	0.01	0.04	0.30	0.767	−0.07 to 0.10
Late trend, *β*_1_ + *β*_3_	−0.02	0.03	−0.63	0.533	−0.09 to 0.05

## 4 Discussion

We are the first to investigate the effects of the DRG reform on medical costs and length of stay for LDH patients in Chinese medicine hospitals. This study provides valuable insights into the ongoing reforms of DRG payment systems in TCM. Our univariate analysis revealed differences in patient characteristics before and after the reform, including sex, age, visit times, admission pathways, admission disease status, complications and comorbidities, and surgeries and operations. Post-reform, there was an increase in female patients, older patients, those admitted through other pathways, and patients with defined disease status, complications and comorbidities, and surgical needs. Notably, the proportion of patients admitted through other pathways and those with repeated hospitalizations also increased, suggesting that the reform measures have successfully attracted more patients and fostered repeat admissions to the hospital. The higher number of patients with defined disease status aligns with the reform’s objective of improving diagnostic precision. Additionally, the increase in patients with complications and operations needs suggests a rise in case complexity, potentially leading to higher reimbursement standards, which benefits the hospital departments during cost settlement.

Integrating the results of multiple linear regression and correlation analyses, we identified significant associations between healthcare-related costs and multiple factors including patient age, sex, complications and comorbidities, visit times, admission pathways, admission disease status, use of Chinese medicine diagnostic and therapeutic equipment, use of Chinese medicine diagnostic and treatment techniques, diagnosis and treatment based on Chinese medicine evidence, and DRG reform. Comprehensive analysis revealed that advanced age, presence of complications and comorbidities, and no use of Chinese medicine diagnostic and therapeutic equipment and use of Chinese medicine diagnostic and treatment techniques were associated with higher hospitalization costs, while DRG reform demonstrated cost-containment effects. Furthermore, a positive correlation was observed among healthcare-related costs and length of stay, suggesting that effective cost control for LDH patients requires comprehensive medication cost management coupled with appropriate reduction of length of stay.

The results from our interrupted time-series analysis indicate a significant downward trend in hospitalization cost, Chinese medicine cost, and Western medicine cost following the DRG reform. While the rising trend in diagnostic cost before the reform was curtailed, overall medical service cost increased post-reform, alongside a marked rise in TCM treatment cost. It is noteworthy that hospitalization cost for LDH patients changed significantly after the DRG reform, demonstrating a marked decrease that aligns with findings from various other studies (41, 43, 44). Certainly, the component costs of hospitalization, including those for Chinese and Western medicine, have concurrently declined alongside the overall reduction in hospitalization cost. However, diagnostic cost and medical service cost were higher post-reform than before, indicating that Chinese hospitals are leveraging their unique strengths by providing more specialized services to address patient needs. This aligns with the Chinese government’s objectives for DRG reform in TCM.

Interestingly, the length of stay for LDH patients did not decrease following the DRG reform, which stands in contrast to findings from several other studies (32, 45–47). What’s more, previous studies suggest that length of stay is a critical factor influencing hospitalization cost, implying that a significant cost reduction should typically accompany shorter stay (48–50). The absence of a reduction in length of stay, despite the cost reduction, may be related to the unique characteristics of TCM treatment, which often has a slower onset of effects and fewer side effects. The relatively prolonged treatment duration for LDH in TCM is inherently difficult to substantially reduce, which is fundamentally associated with TCM’s diagnostic and therapeutic paradigm. First, this stems from TCM’s holistic therapeutic philosophy that necessitates simultaneous management of both pain symptoms and underlying pathological mechanisms (e.g., kidney deficiency, qi-blood stagnation). Second, it reflects the characteristic therapeutic modalities of TCM: acupuncture requires cumulative sessions to activate the endogenous analgesic system, while Chinese herbal compounds need time for multi-target regulation of inflammation and tissue repair. Third, TCM manual therapies follow the natural tissue repair cycle requirements. Although TCM demonstrates a relatively slower onset of therapeutic effects, clinical evidence confirms its superior medium-to-long-term efficacy for LDH management. The majority of TCM treatment occurs in the initial days of hospitalization, suggesting that later cost may be lower, making it challenging to significantly reduce recovery times through DRG payment reforms alone. However, integrating and standardizing TCM treatment methods, along with reducing medication usage, could help achieve the goal of lowering hospitalization cost.

In conclusion, the reform of DRG payment system in TCM has a substantial impact on controlling overall medical costs for LDH patients, although its effectiveness in reducing the length of stay remains limited. Future refinements of the DRG payment system should account for the distinctive characteristics of TCM, optimizing treatment methods and enhancing the quality of healthcare. Establishing a unique and effective reimbursement framework for TCM will further reduce patient treatment cost and length of stay, ultimately benefiting a greater number of patients.

## 5 Conclusion

DRG reform is associated with lower hospitalization cost, as well as reduced costs for both Chinese and Western medicine among LDH patients in Chinese medicine hospitals. However, it does not correlate with reductions in medical services cost, diagnostic cost, TCM treatment cost, or length of stay. While the reform effectively leverages the unique strengths of TCM to alleviate the economic burden of illness, there is a need to deepen institutional reforms and enhance the quality and content of medical services, with the goal of further reducing healthcare costs and shortening hospitalization durations.

## Data Availability

The original contributions presented in the study are included in the article/Supplementary material, further inquiries can be directed to the corresponding author.
